# Evaluation of the Comparative Efficacy of Aquatherapy Versus Conventional Physiotherapy on Motor Function and Psychosocial Well-Being in Children With Acute Lymphoblastic Leukemia: Protocol for a Randomized Controlled Trial

**DOI:** 10.2196/75877

**Published:** 2025-10-23

**Authors:** Shrutika Sharad Khairnar, Sharath Hullumani, Neha Umale

**Affiliations:** 1 Faculty of Paedaitrics and Neonatal Physiotherapy Datta Meghe Institute of Higher Education and Research Wardha India

**Keywords:** acute lymphoblastic leukemia, aqua therapy, rehabilitation, conventional physiotherapy, motor functions, psychosocial well-being, chemotherapy

## Abstract

**Background:**

Children with acute lymphoblastic leukemia (ALL) undergoing chemotherapy have limited access to rehabilitation support. While rehabilitation on the basis of physiotherapy has been found to enhance psychosocial well-being and motor performance, a viable substitute for this can be aquatherapy. However, no trials have currently evaluated the effectiveness of aquatherapy in this population.

**Objective:**

We aim to evaluate the comparative efficacy of aquatherapy versus conventional physiotherapy on the motor function and psychosocial well-being of children with ALL.

**Methods:**

We will conduct a parallel, single-blinded (participant-blinded), randomized controlled trial with embedded quantitative analysis. In total, 54 survivors of ALL undergoing chemotherapy will be recruited from the Acharya Vinoba Bhave Rural Hospital in Sawangi (Meghe), Wardha, Maharashtra, India. The experimental group will attend a 4-week, thrice-weekly, 45-minute aquatherapy session focusing on exercise-based rehabilitation. The comparison group will receive conventional physiotherapy including standardized exercises. Assessments will be completed at weeks 0 (baseline), 4 (postintervention), and 12 (follow-up). The primary outcome related to motor function will be measured using the timed up and go test and the Pediatric Balance Scale at week 4. Secondary outcomes will include psychosocial well-being measured with the Hospital Anxiety and Depression Scale and functional abilities measured with the Functional Independence Measure for Children (WeeFIM). Health service data, including length of stay in the hospital, hospital readmissions, and emergency department presentations, will be recorded. Semistructured interviews will be completed within an interpretive description framework to explore participant descriptions. The primary outcome will be analyzed using linear mixed effects models.

**Results:**

The trial is scheduled to commence in October 2025.

**Conclusions:**

The trial will inform future implementation of rehabilitation for children with ALL by providing important data regarding the efficacy of aquatherapy compared with conventional physiotherapy as well as patient experience.

**Trial Registration:**

Clinical Trials Registry - India CTRI/2025/03/083036; https://tinyurl.com/v446cd6z

**International Registered Report Identifier (IRRID):**

PRR1-10.2196/75877

## Introduction

Acute lymphoblastic leukemia (ALL) is one of the most common cancers affecting children, with about 64,200 cases reported worldwide. In India, the highest incidence occurs among children aged 2–5 years, with rates of approximately 101.4 cases per million boys and 62.3 cases per million girls [1]. The availability of more advanced therapeutic agents, enhanced supportive care, therapy stratification, and regimen modification in recent times has led to a cure rate of more than 80% and an overall survival probability of 80% to 90%. As a result, awareness about therapy-related morbidity has increased because of the longer survival rates [1].

Acute chemotherapy comprises 2 phases: the induction phase and the consolidation phase. The primary goal of the induction phase is remission. Intense chemotherapy is used during this phase, which typically lasts 4 weeks, to destroy as many leukemia cells as possible. After a successful induction therapy session, patients are moved into the consolidation phase, sometimes referred to as intensification. This phase is aimed at eradicating any leukemia cells that could have remained unaffected during the induction phase. Additional chemotherapy regimens that are different from those used in induction are part of the consolidation phase, which usually lasts many months. This is the most intense phase, requiring a prolonged hospital stay. Children with ALL are considerably less active during the acute stage, usually presenting with deficits in fatigue and activity tolerance [2,3]. They may also exhibit motor deficits as a side effect of the chemotherapy drugs, which include anthracyclines and vincristine. While anthracycline-induced cardiotoxicity reduces aerobic capacity, vincristine-induced neuropathy impairs peripheral sensory and motor function [4,5]. Chemotherapeutic drugs such as vincristine have an adverse effect on balance, which may occur through diverse mechanisms such as by decreasing muscular strength and flexibility or producing cognitive impairment [1]. When used on children with ALL, these drugs can result in self-imposed or medically prescribed activity restrictions, which would lead to routinely engaging in lesser physical activity [1,6].

Children with ALL frequently struggle with motor performances, functional movement, and balance, possibly because of deficits in somatosensory, motor, muscular, and cognitive abilities. Numerous studies on children with ALL have examined the effect of exercise regimens on increasing their ankle range of motion, leg and back muscle strength, and cardiovascular fitness [1,7]. However, studies on the impact of therapies aimed at enhancing balance and functional mobility are limited. Furthermore, not enough research has examined their impact during the induction phase, or the acute stage, of chemotherapy. To avoid additional issues related to the disease, physiotherapy interventions conducted in the early stages of the disease can be crucial [1,8]. Such improvements are essential not only for their physical health but also for their psychological well-being, as enhanced mobility can lead to increased participation in daily activities and social interactions.

Exercise in an aquatic environment is part of aquatherapy, or what is known as aquatic treatment; this form of therapy has in recent times drawn much interest as a possible supplement to traditional physiotherapy. The characteristics of water, like buoyancy and resistance, makes it a workout surface that is both supportive and demanding, potentially improving motor function without placing undue strain on the musculoskeletal system. Aquatherapy has demonstrated beneficial benefits on gross motor function and produced enjoyment of physical activity in pediatric groups [9]. In addition to standard physical treatment, aqua-plyometric (plyo) exercises can help long-term survivors of childhood ALL strengthen their bones, increase their functional ability, and enhance their quality of life [10]. One study examined the impact of aquatherapy on performance in children with brain tumors and found that aquatherapy could help their physical performance [11]. However, aquatherapy’s efficacy, especially in children with ALL, remains underexplored.

The primary aim of this randomized controlled trial is to determine the comparative efficacy of aquatherapy versus conventional physiotherapy on motor function in children with ALL during the induction and consolidation phase of their chemotherapy treatment. The secondary aims include determining the comparative efficacy of aquatherapy versus conventional physiotherapy on the (1) psychosocial well-being and (2) quality of life of children with ALL during the induction and consolidation phase of their chemotherapy treatment.

## Methods

### Study Design

**Figure 1:**
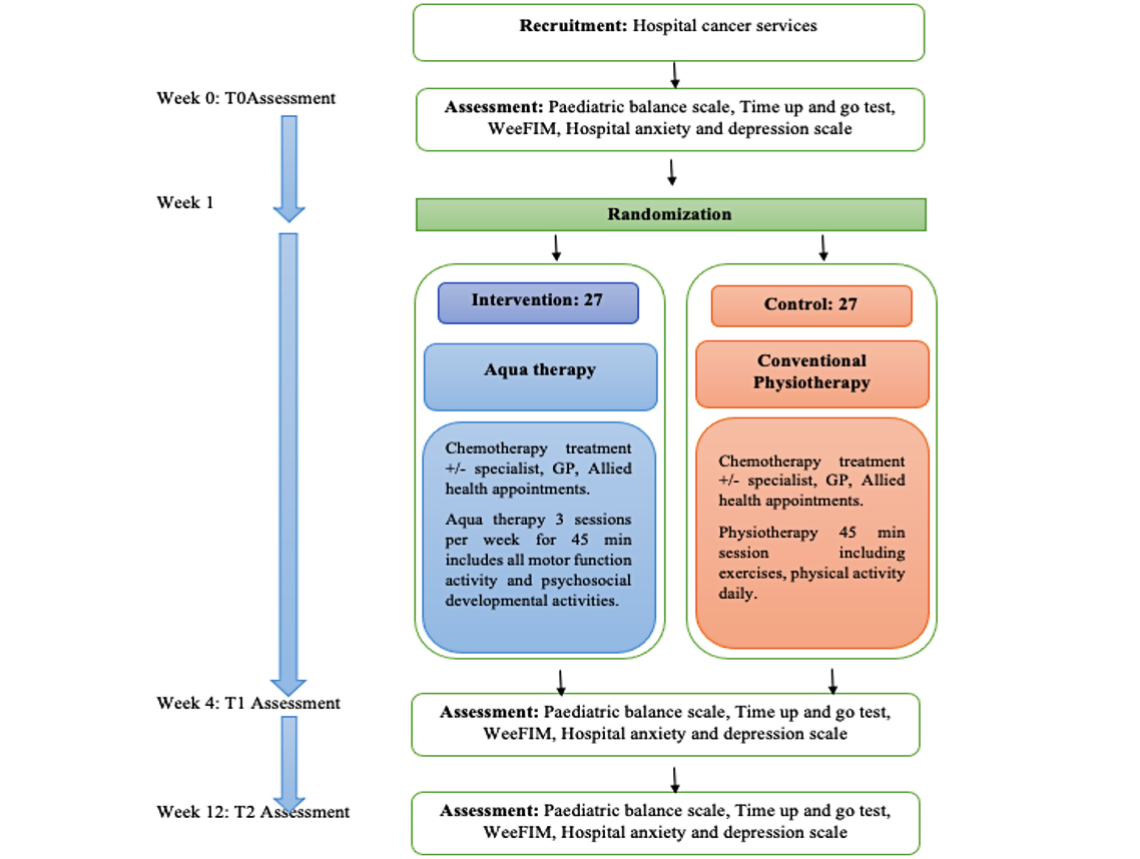
CONSORT diagram for aquatherapy and conventional physiotherapy. GP: general practitioner.

We propose conducting a parallel, single-blinded (participant-blinded), randomized controlled trial with embedded quantitative analysis. The experimental group will attend a 4-week, thrice-weekly, 45-minute aquatherapy session focusing on exercise-based rehabilitation (Figure 1). The comparison group will receive conventional physiotherapy, including standardized exercises. Assessments will be completed at weeks 0 (baseline), 4 (postintervention), and 12 (follow-up). Quantitative trial outcomes will be reported in accordance with the CONSORT (Consolidated Standards of Reporting Trials) statement and SPIRIT (Standard Protocol Items: Recommendations for Intervention Trials) checklist (Multimedia Appendix 1) [12].

On the basis of the acute phase of physiotherapy interventions after chemotherapy treatment in pediatric oncology settings, a 12-week follow-up period is chosen. Clinical trials evaluating early functional and psychosocial responses in children with ALL frequently use shorter follow-up periods. Intensive chemotherapy and related side effects are predicted to cause rapid changes in emotional and physical conditions throughout active treatment phases. Without placing excessive burden on children who are already receiving critical medical care, a 12-week timeline enables the capture of early and clinically meaningful gains.

### Randomization Procedure

Eligible participants who have fulfilled baseline measurements will be randomly allocated to the aquatherapy group or conventional physiotherapy control group using a concealed method in accordance with a web-based computer-generated randomization program using permuted blocks of participants. Allocations will be prepared before trial commencement by an independent researcher with no role in participant recruitment, trial administration of intervention delivery, or assessments. The trial coordinator will allocate participants after baseline assessment by contacting the independent researcher via email for random group allocation.

### Setting

The trial will be conducted at the Acharya Vinoba Bhave Rural Hospital in Sawangi (Meghe), Wardha, Maharashtra, India, a hospital that offers treatment for ALL survivors. Participants will be recruited from the same hospital.

### Ethical Considerations

The aquatherapy trial has been approved by the Datta Meghe Institute of Higher Education and Research (Deemed to Be University), formerly known as Datta Meghe Institute of Medical Sciences (Deemed to Be University) (ECR/440/Inst /MH/2013/RR-2019 DHR; EC/NEW/INST/2023/MH/0340; DMIHER(DU)/IEC/2025/634) (Multimedia Appendix 2), and is registered under the ICMR-National Institute of Medical Statistics Clinical Trial Registry - India CTRI/2025/03/083036 (Multimedia Appendix 3).

### Patient Selection and Consent

Eligible participants will be identified by a physiotherapist. For aquatherapy, the potential participants will be advised about the trial by the clinical staff. If a participant consents, and the parents provide their consent as well (Multimedia Appendix 4), the participant will then be assessed by the physiotherapist. Details of the trial and the aquatherapy session will be provided so that any questions that participants or their parents have can be clarified and written informed consent can be obtained.

### Inclusion and Exclusion Criteria

Participants who are aged 4 to 18 years [1], have ALL, and are undergoing acute stages of chemotherapy treatment can be included. The children must be ambulatory, can be of either sex, and must be in the induction or consolidation phase of chemotherapy for ALL.

Children (1) who are medically unfit to participate in the exercise session as determined by a physiotherapist or medical practitioner considering published recommendations [13]; (2) who have discontinued chemotherapy; (3) who have been diagnosed with sepsis; (4) who have preexisting cardiovascular disease, acute or chronic respiratory disease, acute or chronic bone, joint, or muscular disorders, neuromotor deficits or genetic disorders; and (5) with auditory or visual impairments are excluded from the trial.

### Intervention

All participants will receive their usual medical care, which may include adjuvant, neoadjuvant, or palliative treatment; specialist, nursing, and other health outpatient appointments (eg, to see a physiotherapist); and visits to their general practitioner.

As a part of the trial, participants will not receive standardized hospital educational materials; however, they will be guided solely through supervised aquatherapy sessions.

A conventional physiotherapy session involves motor function and psychosocial developmental exercises with very little support in comparison with aquatherapy. As a part of the trial, conventional physiotherapy also includes a session for 45 minutes, 5 times weekly, including strength training thrice a week [7,14,15]. All participants will also have the opportunity to discuss their ongoing rehabilitation needs at the end of the 4-week intervention period. They will be provided written information for referral to appropriate local services for ongoing support if required in line with usual practice at the health service.

### Experimental Group: Exercise-Based Aquatherapy

In addition to conventional physiotherapy, participants randomized to the experimental group, which involves aquatherapy, will receive a 45-minute exercise session delivered by the physiotherapist in the aquatic machine thrice weekly for 4 weeks. The exercises include aerobic training, psychosocial developmental well-being, and resistance training guided by published recommendations [7,14,15]. These exercise sessions will be individually tailored and include a waterjet in the aqua machine for resistance, treadmill walking in the aqua machine, balance activities, aerobic training, and functional activities. All these exercises, which are part of endurance training, will be conducted within the aquatherapy machine itself, involving mild to moderate aerobic exercises (eg, slow paced treadmill walking in the aqua machine, water-splashing activity, spot marching, and sidestepping). The aquatherapy machine also has grab bars and a seat for exercises that work on improving balance. The therapist will choose exercise variations, for example, upper-limb strengthening by using weights or lower-limb strengthening by using the waterjet provided in the aqua machine. Initial warm-up exercises include various upper- and lower-body stretches and balance exercises. Water-splashing activities with play therapy is included to improve psychosocial well-being. Exercise intensity will depend on verbal feedback from the participant, observation by the physiotherapist, and as per standard protocol-based intensity. Resistance exercise will involve, first, active-assisted range of motions followed by active range of motions. To improve balance, aqua-plyometric exercises will also be done in the aqua machine under the physiotherapist’s guidance. Before starting the exercises, aerobic training as a warm-up for 5 minutes will be followed by a cool-down for 5 minutes (Table 1).

The participants will receive an overview of the exercise regimen and study protocols linked to rehabilitation from the physiotherapist who will be evaluating and administering the aquatherapy intervention. The sessions will be for 45 minutes each, with 3 sessions per week for 4 weeks [10,11]. Activities are aimed at improving motor function and psychosocial well-being. The fidelity of the intervention will be monitored by recording the content of the exercise session and duration of the completed sessions. Participants in both groups will also be asked whether they received any exercise-based intervention outside of the trial at the 4-week assessment and follow-up.

**Table 1:** Intervention and comparison group descriptions based on the TIDieR (Template for Intervention Description and Replication) checklist.

	Experimental group	Comparison group
In brief	Aquatherapy rehabilitation	Conventional physiotherapy
Why	Exercise intervention in the aquatherapy machine is safe, effective, and convenient to use for improving the quality of life of individuals with ALL undergoing chemotherapy.	Individuals with ALL undergoing chemotherapy are routinely offered rehabilitation.
What (materials)	Preparations before the aquatherapy session: Avoid a heavy meal before the session Remain well hydrated before the session Maintain water temperature Wear proper clothing before entering the aquatherapy machine Follow general principles for duration, frequency, and intensity to be maintained Provide participant with weight cuff Aquatic therapy machine equipped with an underwater treadmill and adjustable resistance jets	Rehabilitation is delivered using standard physiotherapy techniques without the use of any specialized equipment Rehabilitation focuses on land-based exercises
What (procedures and heath care provider)	Physiotherapist will provide aquatherapy session protocol in written format	Physiotherapist will provide the exercise guidelines
How	Supervised session in aquatherapy machine Digitally controlled aqua machine allows for real-time monitoring of water speed and session duration, ensuring consistency in exercise intensity.	In person
Where	Aquatherapy machine is placed in the pediatric outpatient department Participants receive aquatherapy in the pediatric outpatient department	Participants receive conventional physiotherapy in the hospital (inpatient department) itself
When and how much (intensity)	Warm up: stretching 30 s, hold, 3 repetitions Aerobic exercises: underwater treadmill walking with minimum speed Strengthening exercises: 10 repetitions, 2 sets Resistance exercises: 10 repetitions, 2 sets Balance exercises: 5 s, hold single leg stance, and progress up to 10 s holds	Fatigue: Jacobson relaxation exercise and aerobic exercises like walking Strengthening exercises: 10 repetitions, 3 sets Resistance exercises: 10 repetitions, 2 sets Balance exercises – 5 s hold single leg stance and then progress up to 10 s holds (eg, heel walking, toe walking, with eyes open and eyes closed)
Frequency	3 sessions per wk, supervised	5 sessions per wk, supervised
Session time	45 min	45 min
Overall duration	4 wk	4 wk
Tailoring	Exercise is tailored to each participant’s ability	Exercise is tailored to each participant’s ability
Trial fidelity	Physiotherapist Intervention will be delivered as planned Ensuring all participants receive the intervention in the same way	Physiotherapist Intervention will be delivered as planned Ensuring all participants receive the intervention in the same way

### Study Outcomes

Participants will be assessed at weeks 0, 4, and 12. The primary end point is immediately after the intervention (week 4). Physical activity, balance, and psychosocial well-being will be assessed at weeks 0 and 4. Primary and secondary outcomes are as outlined in Table 2.

**Table 2:** Primary and secondary outcomes, measures, and time points of the intervention.

Outcomes	Measures or sources	Definitions	Time points
Primary outcome: balance	Pediatric Balance Scale [16]; reliability 0.97	This 14-item scale is simple to administer, does not require any specialized equipment. Performance can be measured both quantitatively and qualitatively using a 0 to 4 grading system.	Wk 0; wk 4
Functional mobility	Timed up and go test [1,17]; reliability (ICC>0.80)	The test evaluates functional mobility and balance by timing how long it takes a participant to get up from a seated posture, walk 3 min, turn around, go back, and sit down. <10 s—typical mobility; 10-14 s—independent walking but modest motor deficits. >20 s—severe restrictions with higher risk of fall; 15-20 s—intermediate mobility issues, requiring some assistance	Wk 0; wk 4
Secondary outcome: motor function	Functional Independence Measure for Children (WeeFIM) [18,19]	A popular instrument for evaluating the functional capacities of individuals undergoing rehabilitation, the functional independence measure focuses on independence in everyday activities. Each of the 18 items in the FIM is scored on a scale of 1–7, with higher scores indicative of greater independence. The items are separated into motor and cognitive categories. Development and rehabilitation requirements of individuals can be inferred from their overall score, which ranges from 18 (total reliance) to 126 (full independence).	Wk 0; wk 4
Psychosocial well-being	Hospital Anxiety and Depression Scale (supplementary file [18])	It is a 14-item questionnaire used in hospital settings to measure sadness and anxiety. Each of the two subscales (depression and anxiety) has 7 items and is scored between 0 and 3, with a maximum score of 21 per subscale; 0-7 (normal), 8-10 (mild), 11-15 (moderate), and 16-21 (severe) are the interpretations for the scores. The HADS^a^ is a useful instrument for early detection and monitoring of mental health issues in individuals receiving therapy because a score above 8 suggests substantial symptoms.	Wk 0; wk 4

^a^HADS: Hospital Anxiety and Depression Scale.

“Adverse events,” as defined by the World Health Organization [19], will be recorded from medical records, direct observation during classes, and by participants’ self-reporting to document the safety of the intervention. The event may or may not be related to the intervention but may occur while the individual is participating in the intervention phase of the trial. Adverse events will be categorized as minor or serious, related or unrelated, and expected or unexpected. A minor adverse event is defined as an incident that occurs during the intervention but results in no injury or minor injury requiring no or minor medical intervention. A serious adverse event is defined as an incident that occurs during the intervention, resulting in death, serious injury, or hospitalization (eg, injurious fall resulting in fracture). Serious adverse events will also be recorded for the usual care group. In addition, adverse events will be reported and graded using the Common Terminology Criteria for Adverse Events Version 4 [20]. The consequences of a serious adverse event in the control group (eg, hospitalization and emergency-department admission) will be captured by the health service utilization questionnaire and medical record audit. Reasons for nonparticipation in an exercise session or noncompletion of the program will also be recorded (eg, pain, fatigue, and feeling unwell).

### Participant Safety and Ethical Considerations

The quality of the water will be routinely observed and recorded. Until medically cleared, children with open wounds, central lines, or symptoms of skin infections will not be allowed to participate in aquatherapy. Before each session, a pediatric oncologist will check all participants for symptoms of fever or active infections. For emotional support during the first sessions, parents or other caregivers may be present. The research ethics board will be notified of all adverse events, mild or major. Before participating, families will be briefed on any possible dangers and asked to sign a form providing informed consent and assent. Periodically, a data safety monitoring team will examine events and make necessary protocol adjustments.

### Consumer Perceptions

Semistructured interviews will be conducted twice before the intervention (week 0) and after the intervention (week 4), with participants in the aquatherapy group to explore their experiences with the program. A purposive sample of participants will be asked about satisfaction, perceived benefits, and the ease of participating in aquatherapy. The same group will be interviewed at both time points. Interviews will continue until data saturation is reached and no new themes emerge.

### Sample Size Estimation

It is estimated that 54 participants will be required [1].

Sample size calculation for paired means (before-after) functional mobility.

The following formula was used:

n ≥ [2(Z_1–α/2_ + Z_1–β_)^2^ / (δ_diff / σ_diff)^2^] + Z_1–α/2_^2^ / 2

Z_1–α/2_ = 1.96

Z_1—β_ = 1.64 at 95% power

The mean difference (δ_diff) was 1.12 before and after the results as per the reference article. The SD (σ_diff) was 1.09.

n = 2 × (1.96 + 1.64)^2^ / (1.12 / 1.09)^2^ = 24.55 = (1.96^2^) / 2 = 1.92

The sample size was 26.47≈27, and the minimum paired sample size needed was 27 for each group.

### Statistical Analysis: Analysis of Quantitative Data

All statistical analyses will be conducted using SPSS (IBM Corp) or equivalent statistical software. Descriptive statistics will be used to summarize demographic variables and baseline characteristics. Continuous variables such as scores from the Pediatric Balance Scale and Aquatherapy (primary outcomes) as well as Functional Independence Measure for Children (WeeFIM) and Hospital Anxiety and Depression Scale (secondary outcomes) will be expressed as mean (SD). To assess within-group changes (before vs after the intervention), paired 1-tailed *t* tests will be applied if data are normally distributed; otherwise, Wilcoxon signed-rank tests will be used. For between-group comparisons (aquatherapy vs conventional physiotherapy), an independent *t* test will be used for normally distributed data, and Mann-Whitney *U* tests will be used for nonparametric distributions. In addition, the analysis of covariance may be used to adjust for any baseline differences. A *P* value of <.05 will be considered statistically significant.

## Results

The trial has not yet commenced and will commence in October 2025.

## Discussion

### Anticipated Findings

The functional mobility and balance of children receiving therapy for ALL can be improved by a tailored intervention, as per this study. Our results could be consistent with earlier studies that demonstrate the advantages of virtual reality and exercise-based therapies in pediatric oncology rehabilitation [1]. Rehabilitation techniques have been found to improve balance and motor function impairment resulting from chemotherapy-induced neurotoxicity, such as vincristine-induced peripheral neuropathy [5]. Physical activity during cancer therapy has been found to reduce some of the adverse effects of ALL treatments [6]. Furthermore, research on the effects of exercise programs, such as aquatherapy and plyometric exercises, has shown that children who have survived cancer have significantly increased bone mineral density and functional capacity [13]. This study proposes that children receiving treatment for ALL may benefit in terms of physical health from structured movement-based therapy.

These findings have clinical relevance as they support the value of structured rehabilitation programs in pediatric oncology settings. Prior research has highlighted the need for incorporating exercise therapies into standard cancer care to improve overall survival and quality of life [13]. The study suggests how aquatherapy can improve motor function, balance, and general physical activity in this susceptible group when the intervention is conducted in the induction and consolidation stages of treatment. Exercise therapies have been shown to dramatically improve physical results in children with ALL, and aquatherapy may provide extra advantages because of its supportive setting, which promotes mobility while reducing joint stress. This study may prove beneficial for developing evidence-based rehabilitation plans that improve the functional recovery and quality of life for young patients with ALL.

### Strengths and Limitations

For pediatric patients, virtual reality–based rehabilitation offers an interesting and feasible approach, much like research that uses interactive and technology-assisted exercise regimens [8-10]. The results highlight the importance of rehabilitation techniques in standard cancer care and add to the body knowledge on physical activity therapy in ALL patients [11-14]. To ensure reliable data collection and analysis, our study used validated mobility and balancing measurements. This study backs earlier findings about the advantages of exercise-based therapies for childhood malignancies, including ALL [15-17].

Our findings might not be as applicable to a larger group of pediatric patients with ALL owing to the small sample size. The immediate postintervention results were the main focus of our investigation; long-term advantages or the sustainability of gains were not evaluated [18,19]. Several variables that could affect the results of the rehabilitation were not considered, including variations in chemotherapy regimens, dietary state, and psychosocial support [20-25].

### Conclusions

Aquatherapy is a rapidly growing area that may have many positive impacts among individuals with ALL undergoing chemotherapy. This trial has the potential to inform future models of cancer rehabilitation mainly aquatherapy intervention, which can be implemented in health services to improve access to aquatherapy for patients with ALL.

## Appendix

Multimedia Appendix 1 SPIRIT (Standard Protocol Items: Recommendations for Intervention Trials) checklist.

Multimedia Appendix 2 Institutional ethics committee certificate.

Multimedia Appendix 3 Clinical Trial Registry India registration.

Multimedia Appendix 4 Informed consent form.

## Abbreviations

ALL: acute lymphoblastic leukemia
CONSORT: Consolidated Standards of Reporting Trials
SPIRIT: Standard Protocol Items: Recommendations for Intervention Trials
WeeFIM: Functional Independence Measure for Children
