# Supplementary material for: Evaluation of the Comparative Efficacy of Aquatherapy Versus Conventional Physiotherapy on Motor Function and Psychosocial Well-Being in Children With Acute Lymphoblastic Leukemia: Protocol for a Randomized Controlled Trial
Source: JMIR Res Protoc. 2025 Oct 23;14:e75877. doi: 10.2196/75877 (PMC12592890; doi:10.2196/75877)
Supplement: Multimedia Appendix 2 [file resprot_v14i1e75877_app2.pdf]

DATTA MEGHE INSTITUTE OF HIGHER EDUCATION & RESEARCH
[DEEMED to be UNIVERSITY]
Formerly known as Datta Meghe Institute of Medical Sciences (Deemed To Be University)
Re-accredited with NAAC Grade A++

INSTITUTIONAL ETHICS COMMITTEE

DCGI Re-regd. No. ECR/440/Inst/MH/2013/RR-2019
DHR Regd. No. EC/NEW/INST/2023/MH/0340
Sawangi (Meghe), Wardha-442 107, Maharashtra, India
Ph. No. : 07152 - 287701, 287702, 287703, 287704, 287705, 287706, 254501
Email :- iec.dmiher@gmail.com, Website : www.dmiher.edu.in

Ref.No. DMIHER(DU)/IEC/2025/634

Date: 08/02/2025

CERTIFICATE OF APPROVAL

The Institutional Ethics Committee in its meeting held on **30th January 2025** has approved the following research proposed to be carried out at Ravi Nair Physiotherapy College, Datta Meghe Institute of Higher Education and Research (DU), Sawangi (Meghe), Wardha.

This approval has been granted on the assumption that the proposed research work will be carried out in accordance with the ethical guidelines prescribed by Central Ethics Committee on Human Research (C.E.C.H.R.)

The details of the proposed research project for dissertation work of postgraduate program in Master of Physiotherapy (MPT) degree course discussed and approved by Institutional Ethics Committee, DMIHER(DU) are as under:-

**Name of the student** Research worker	**Name of Guide** Guidance/Supervision	**Name of Instt.** Name of Dept.	**Title of the proposed research** (PG thesis topic)	**Category** (Specialization) in subject	Approval Status
**Dr. Shrutika Khairnar**	Guide/supervisor<br>Dr. Neha Umale<br><br>Co-guide<br>Dr. Sharath Hullumani	**RNPC**<br>Dept. of Pediatric Physiotherapy	Comparative Efficacy of Underwater Rehabilitation Versus Conventional Physiotherapy on Motor Impairment in Children with Acute Lymphoblastic Leukemia: A Randomized Controlled Trial.	**PG-Thesis** for [Master of Physiotherapy]<br><br>**MPT** in **(Paediatrics Physiotherapy)**	Approved

(Dr. Swanand Pathak)
Member Secretary
Institutional Ethics Committee
DMIHER (DU)

D.M.I.H.E.R. (D.U.)
APPROVED
INSTITUTIONAL ETHICS COMMITTEE

Copy to :-

1. Dr. Shrutika Khairnar, MPT student
2. Dr. Neha Umale, Asso.Professor and Guide, Dept. of Pediatrics Physiotherapy, RNPC
3. Dr. Sharath Hullumani, Asst.Professor and Co-guide, Dept. of Pediatrics Physiotherapy, RNPC
4. Principal, R.N. Physiotherapy College

Created with cam scan
