# Supplementary material for: Evaluation of the Comparative Efficacy of Aquatherapy Versus Conventional Physiotherapy on Motor Function and Psychosocial Well-Being in Children With Acute Lymphoblastic Leukemia: Protocol for a Randomized Controlled Trial
Source: JMIR Res Protoc. 2025 Oct 23;14:e75877. doi: 10.2196/75877 (PMC12592890; doi:10.2196/75877)
Supplement: Multimedia Appendix 3 [file resprot_v14i1e75877_app3.pdf]

Clinical Trials Registry - India (ICMR-NIMS)

SOP to be followed for field unlocking in registered trials

For site addition/deletion - Please upload EC/DCGI approval of additional site or site deletion under Ethics Approval - this field is permanently unlocked and revert by mail for site unlocking. Please also mention he list of new site PI in the mail. For those sites which have not received EC approval, please mark a copy of the mail to the PI requesting a confirmation email to this email ID regarding their participation in trial.

For new contact person (Overall trial PI/Scientific/public query) - Please indicate new person, mark a copy of the mail to concerned person and request mail confirmation of responsibility

For Intervention/comparator agent/ inclusion & exclusion criteria, sample size, scientific title primary and secondary outcome, please specify changes (in a tabular format) and confirm if EC approval has been received for the same, if applicable, and upload in EC section which is permanently unlocked.

Registered Trials

otal Number of Registered Trials=1

CTRI Reg. Date	CTRI Reg. No	Reference No.	Type of Trial	DCGI Clearance	EC Clearance	Recruitment Status India	Modification	Details
21/03/2025	CTRI/2025/03/083036	REF/2025/03/101803	Interventional	Not Applicable	Approved	Open to Recruitment	Click	Click to View Details
