# Supplementary material for: Evaluation of the Comparative Efficacy of Aquatherapy Versus Conventional Physiotherapy on Motor Function and Psychosocial Well-Being in Children With Acute Lymphoblastic Leukemia: Protocol for a Randomized Controlled Trial
Source: JMIR Res Protoc. 2025 Oct 23;14:e75877. doi: 10.2196/75877 (PMC12592890; doi:10.2196/75877)
Supplement: Multimedia Appendix 4 [file resprot_v14i1e75877_app4.pdf]

CONSENT FORM

INFORMED CONSENT FORM

Title of the Project: **Evaluation of Comparative Efficacy of Aqua-therapy Versus Conventional Physiotherapy on Motor Function and Psychosocial Well Being in Children with Acute Lymphoblastic Leukaemia: Protocol for a Randomised Controlled Trial**

Name of the Investigator – Dr. Shrutika

I have received the information on the above study and have read and /or understood the information. I have been given the chance to discuss the study and ask questions. I consent to take part in the study and I am aware that my participation is voluntary. I understand that I may withdraw at any time without this affecting my future care. I understand that the information collected about me from my participation in this research and sections of any of my medical notes may be looked at by responsible persons (ethics committee members / regulatory authorities). I give access to these individuals to have access to my records.

____

Signature / Thumb Impression of subject with date

____

Name of the subject in capitals

INFORMED ASSENT FORM

TITLE: Evaluation of Comparative Efficacy of Aqua-therapy Versus Conventional Physiotherapy on Motor Function and Psychosocial Well Being in Children with Acute Lymphoblastic Leukemia: Protocol for a Randomized Controlled Trial

PURPOSE OF STUDY: The study evaluates the effectiveness of aqua therapy alongside conventional physiotherapy in improving motor function, strength, and rehabilitation in children with acute lymphoblastic leukemia (ALL) during the induction and consolidation phases of treatment. By comparing outcomes between hydrotherapy and physiotherapy-only groups, it aims to determine the benefits of integrating hydrotherapy into rehabilitation protocols. The goal is to mitigate motor impairments caused by ALL treatments and enhance recovery. This research seeks to improve the quality of life and functional capabilities of young cancer patients.

PROCEDURE: You will be recruited in the study only if you fulfil the inclusion criteria. You will be explained about the procedure and its effect before the commencement of the study.

RISKS: There is no risk to you as a result of participation in this study.

PRIVACY AND CONFIDENTIALITY: Privacy and confidentiality of the information you provide will be safeguarded for subject to any legal requirement.

FINANCIAL INCENTIVE FOR PARTICIPATION: You will receive financial assistance as no charges for treatment for participating in this study.

ALTERNATIVES: If you decide to participate in the study, you will be benefited by the program. If you decide not to participate in the study and/ or even wish to drop out any time during the study, conventional physiotherapy will be given to you.

AUTHORIZATION TO PUBLISH RESULTS: Results of this study may be published for scientific purposes and / or presented to scientific groups; however, you will not be identified.

VOLUNTARY PARTICIPATION AND AUTHORIZATION: Your participation in

this study is voluntary. Your decision whether or not to participate in the study will not affect your care during your hospital admission. You are free to discontinue participation in this study at any time and for any reason. In case, you need any further information regarding your rights as study participant you may please contact

STATEMENT OF CONSENT: I, Mr./Ms. ……………………………………………. volunteer and consent to participate in this study. The contents of the study have been fully read and explained to me in vernacular language and the same is true and correct to the best of my knowledge, information and belief. I may ask questions at any time during the study.

……………………..	……………………………
Name of the Participant	Signature/left thumb impression
……………………..	……………………………
Name of the witness	Signature/left<br>thumb impression
……………………..	……………………………
Name of the investigator	Signature of the investigator
